# Effect of Resveratrol on Serum Levels of Type II Collagen and Aggrecan in Patients with Knee Osteoarthritis: A Pilot Clinical Study

**DOI:** 10.1155/2021/3668568

**Published:** 2021-11-11

**Authors:** Bushra Hassan Marouf

**Affiliations:** Department of Pharmacology and Toxicology, College of Pharmacy, University of Sulaimani, 46001 Kurdistan Region, Iraq

## Abstract

Treatment of knee osteoarthritis (OA) remains a challenging concern. Preclinical studies provided accumulating evidence on resveratrol efficacy in ameliorating degenerative articular damage. The present study was conducted to evaluate the effects of resveratrol as monotherapy on the serum level of type II collagen (Coll 2-1) and aggrecan in patients with knee osteoarthritis. The study was an open-labeled noncontrolled clinical trial. Resveratrol 500 mg/day in a single oral dose was given to the patients with knee osteoarthritis for 90 days. The serum levels of Coll-2-1, aggrecan, and biomarkers of inflammation were measured pre- and posttreatment. Hematological profiles and both hepatic and renal function markers were investigated at the baseline and at the end of the treatment for evaluating the tolerability and safety of resveratrol. Visual Analog Scale (VAS) for pain and Knee injury and Osteoarthritis Outcome Score (KOOS) for disease activity were clinically assessed monthly. Administration of 500 mg resveratrol for three months led to a nonsignificant decrease in the serum level of Coll 2-1 while a significant increase in aggrecan serum level. Resveratrol significantly improves pain score measured by VAS and KOOS after 30 days. Improvements in patients' activity and functional status were also evident at day 30 and kept on for three months which was reflected by KOOS subscale scores and with a significant improvement in all KOOS areas. In conclusion, oral administration of resveratrol as a monotherapy provides a remarkable improvement in the clinical status of the patients but has no significant effect on serum levels of Coll 2-1.

## 1. Introduction

Knee osteoarthritis (OA) is a complex degenerative articular disorder that is described by the degeneration of articular cartilage, mostly proteoglycans, resulting in damage of articular tissue and hypocellular outcome that consequently leads to impair joint function [1]. The treatment of knee osteoarthritis remains unresolved. Most conventional therapy concentrates on relieving pain and other complains of the disease [2–4], but their efficacy on the development of the disease is limited, and they are usually accompanied by many adverse effects that encouraged the usage of safer therapy with natural origin [5–9].

Many phytochemicals with pleiotropic effects are affirmed to be chondroprotective agents, and they have been highlighted as alternative therapy for OA. Resveratrol, *trans*-3,5,4-trihydroxystibene, is a natural phytoalexin derived from Polygonum cuspidatum; it is found in high concentration in the grape skin, cranberries, and peanuts. It is considered as a rational herbal candidate with a potential therapeutic interest in joint disorder; it also shows pleiotropic effects. Its anti-inflammatory and OA-protective effects have been documented in many studies [10, 11]. Preclinical studies provided accumulating evidence on resveratrol efficacy in ameliorating the degenerative articular damage. In recent years, a clinical trial exhibited the efficacy of resveratrol as add-on therapy in mitigating the OA pain and lowering the proinflammatory markers [12]. However, clinical trials that explore the efficacy of resveratrol monotherapy in joint disorder such as OA through investigating cartilage-derived molecules are obscure to date.

Seeking for an efficacious and safe treatment for OA is not enough; it also needs to target more specific macromolecules in the articular cartilage to observe the progression of the disease and monitor the efficacy of the treatment. In the past decade, various efforts have been carried out in that direction. A graduated tissue structure of articular cartilage consists of three main components, chondrocyte, collagen type II network, and proteoglycan. In patients with osteoarthritis, Coll 2-1, products of type II collagen network, and proteoglycan (aggrecan is the major protein in cartilage) seem to be dependable and certain biomarkers for OA [13, 14].

For this purpose, the present study has been designed to assess the clinical efficacy of resveratrol as a monotherapy on serum level of both type II collagen (Coll 2-1) “a biomarker of collagen network degradation” and aggrecan “a degradation product of articular cartilage” biomarkers and on the evaluation of pain and OA symptoms in patients with mild and moderate knee osteoarthritis before and after 90-day administration of oral doses of resveratrol.

## 2. Material and Methods

### 2.1. Study Design and Setting

The study was an open-labeled noncontrolled clinical trial; it was carried out in accordance with the Declaration of Helsinki and its amendments with the Ethical Guidelines for Human Studies and the currently adopted regulations of the Iraqi MOH. The protocol of the trial was registered in ISRCTN registry with study ID ISRCTN75392625.

**Figure 1:**
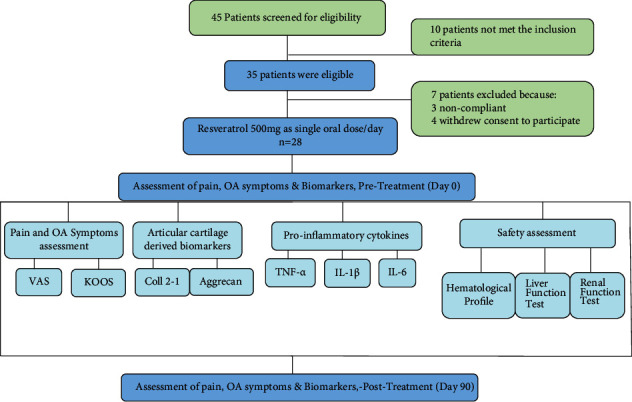
Study design flow chart including patient's enrollment, interventions, parameters of the study, and duration of the trial. OA: osteoarthritis; VAS: Visual Analog Scale; KOOS: Knee injury and Osteoarthritis Outcome Score; Coll 2-1: type II collagen; TNF-*α*: tumor necrosis factor-*α*; IL: interleukin; *n*: number of patients.

The study was conducted at the Shar Teaching Hospital of Sulaimani City, Iraq. Study approval was obtained from the ethical committee of the College of Medicine, University of Sulaimani. All patients have given written informed consent to participate. Eligible patients were selected by senior orthopaedician and rheumatologist based on the American College of Rheumatology criteria (ACR) and the radiographic evidence [15]. The enrolled patients had osteoarthritis (with mild-to-moderate grade). The study was a 90-day administration of oral resveratrol 500 mg/day in a single dose (Figure 1). Resveratrol was prepared as a capsule dosage form (specially prepared for this purpose); it was given by a senior pharmacist with full instruction. Resveratrol was purchased as Trans-Resveratrol natural pure powder ≥ 98% (HPLC on anhydrous basis) (botanical source: *Polygonum cuspidatum* from Apollo Healthcare Resources, Singapore). Acetaminophen 500 mg up to four tablets per day as a rescue medication was allowed to the patients for nontolerated pain they experienced during the study. Duration of the study was 11 months including patient recruitment (which was three months), intervention, follow-up, and laboratory investigations. Patient's compliance was enhanced by phone call communication and asking them to return the empty containers of the tested drugs on every follow-up visit.

### 2.2. Inclusion and Exclusion Criteria

Patients with mild-to-moderate OA as defined by the revised criteria of American College of Rheumatology (ACR) for the diagnosis of knee OA were included. Patients with chronic musculoskeletal disorders such as rheumatoid arthritis and gout, ischemic heart disease, heart failure, chronic kidney disease, and hepatic failure were excluded. Furthermore, patients using nonsteroidal anti-inflammatory drugs or corticosteroid medications four weeks prior to the study and intraarticular injection within 3 months prior to the study recruitment, lactating, and pregnant woman or planned to be pregnant were not included.

### 2.3. Primary Outcome Measure

The serum levels of Coll-2-1 and aggrecan were determined pre- and posttreatment (i.e., at day 0 and at day 90) using an enzyme-linked immunosorbent assay (ELISA) kit (Bioassay Technology Laboratory, Korain Biotech Co., Ltd., Shanghai, China, and DRG International Inc., USA, respectively) according to the manufacturer's instructions. Additionally, biomarkers of inflammation (interleukin- (IL-) 6, IL-1*β*, and tumor necrosis factor-*α* (TNF-*α*)) using enzyme-linked immunosorbent assay (ELISA) kit (Bioassay Technology Laboratory, Korain Biotech Co Ltd., Shanghai, China) according to the manufacturer's instructions were also evaluated twice at baseline at day 0 (pretreatment) and at day 90 (posttreatment).

Standard hematological profile (using the Swelab Alfa Plus system) and liver function test and kidney function markers were carried out spectrophotometrically at baseline and at the end of the treatment using the clinical chemistry analyzer Cobas c 311, utilizing ready-made kits according to the manufacturer's recommendations for evaluating safety and tolerability of resveratrol.

### 2.4. Secondary Outcome Measure

As secondary outcome measures, the Visual Analog Scale (VAS) for pain and Knee injury and Osteoarthritis Outcome Score (KOOS) for measurement of pain and clinical status (ranging from 0 (worst) to 100 (best)) were utilized. Disease activity was clinically assessed at the baseline (day 0) and at 30-day intervals during the study. Assessment criteria are based on changes from baseline observed to the end of month three.

### 2.5. Statistical Analysis

Statistical analysis of results was performed by using GraphPad Prism 8 version 8.4.3 for macOS, (GraphPad Software, San Diego, California USA). For quantitative variables, the data were described as mean and standard deviation (SD), while for the categorized variables, a table of frequency including numbers and percent was utilized. A comparison of pre- and posttreatment results and parameters of the study group was carried out using a paired *t*-test.

## 3. Results

A total of 45 patients were screened for eligibility. Thirty-five patients were eligible for inclusion. Twenty-eight patients completed the 90-day study; seven patients were excluded due to incompliance (3 patients) and withdrawal of consent to participation in the therapy (4 patients).

Twenty (71.5%) of the participants were female, and 8 (28.5%) were male. The mean age of them was 55.96 ± 7.67 years, and the mean body weight was 82.75 ± 15.83 kg. Half of the participants were with mild 14 (50%) grade of OA while the other half was with moderate 14 (50%). At the baseline, the participants reported a high level of knee pain with a mean VAS pain score of 74.32 ± 8.36 and a total KOOS score of 45.69 ± 12.74 (Table 1).

### 3.1. Effect of Resveratrol on Coll 2-1 and Aggrecan Serum Level

**Figure 2:**
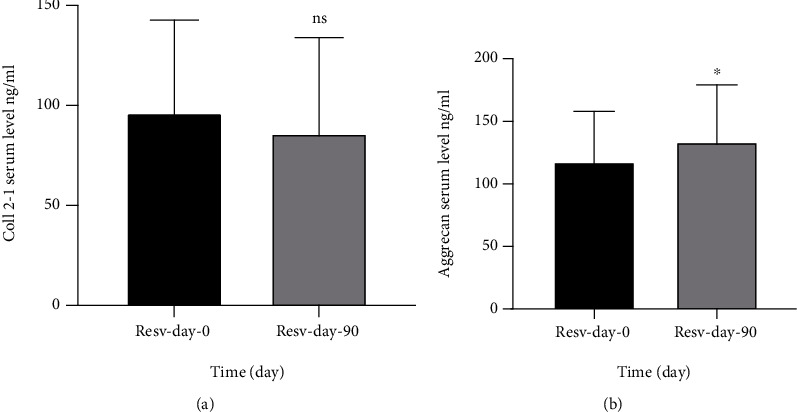
Effect of resveratrol on serum level of (a) Coll 2-1 in patients with knee OA using resveratrol for 90 days and (b) aggrecan in patients with knee OA using resveratrol for 90 days. Resv: resveratrol; Coll 2-1: type II collagen; ns: nonsignificantly different comparison of posttreatment with the baseline (pretreatment) using paired *t*-test (*P* > 0.05). ^∗^ Significantly different comparison of posttreatment with the baseline (pretreatment) using paired *t*-test (*P* < 0.05).

Administration of 500 mg resveratrol for 90 days led to a nonsignificant decrease in the serum level of collagen type II (Coll 2-1) with *P* value > 0.05 (Figure 2(a)), while the result of the aggrecan level shows a significant increase in its serum level (*P* value < 0.05) (Figure 2(b)).

### 3.2. Effect of Resveratrol on Pain and the Clinical Outcome of the Patients

**Figure 3:**
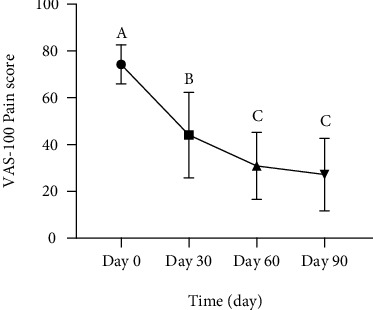
Monthly mean changes of VAS-100 pain score in patients with knee osteoarthritis using resveratrol for 90 days. Values with different superscripts (A, B, and C) are significantly different among different times (using ordinary one-way ANOVA test; *P* < 0.05).

**Figure 4:**
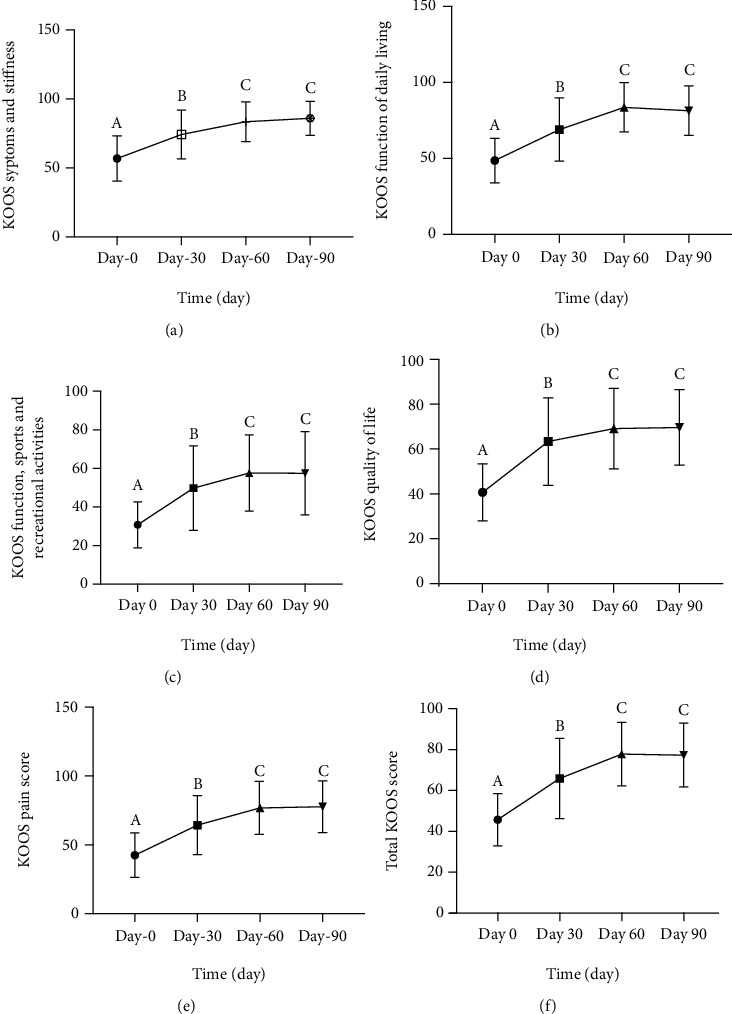
Monthly mean changes of KOOS subscale score (a–e) and total KOOS score (f) in patients with knee osteoarthritis using resveratrol for 90 days. Values with different superscripts (A, B, and C) are significantly different among different times (using ordinary one-way ANOVA test; *P* < 0.05).

As a secondary outcome measure, KOOS total score (ranging from 0 (worst) to 100 (best)) was utilized to assess the clinical outcome of resveratrol. Use of resveratrol was associated with improvement in pain measured by both VAS and KOOS pain scores with statistically significant reductions in both VAS pain scores (*P* value < 0.05) and KOOS pain scores (*P* value < 0.05) that were observed after 30 days, and it continues to improve for 90 days (Figures 3 and 4(e), respectively). Furthermore, improvements in patients' activity and functional status were also evident at day 30, which was reflected by KOOS subscale scores, and there was a significant improvement in all KOOS areas including symptom and stiffness, function of daily living, sports and recreational activities, and quality of life with *P* value < 0.05 (Figures 4(a)–4(d)). There was also a statistically significant improvement in KOOS total scores after 30 days of using resveratrol and continue for the entire period of the study (Figure 4(f)).

### 3.3. Effect of Resveratrol on Proinflammatory Cytokines

**Figure 5:**
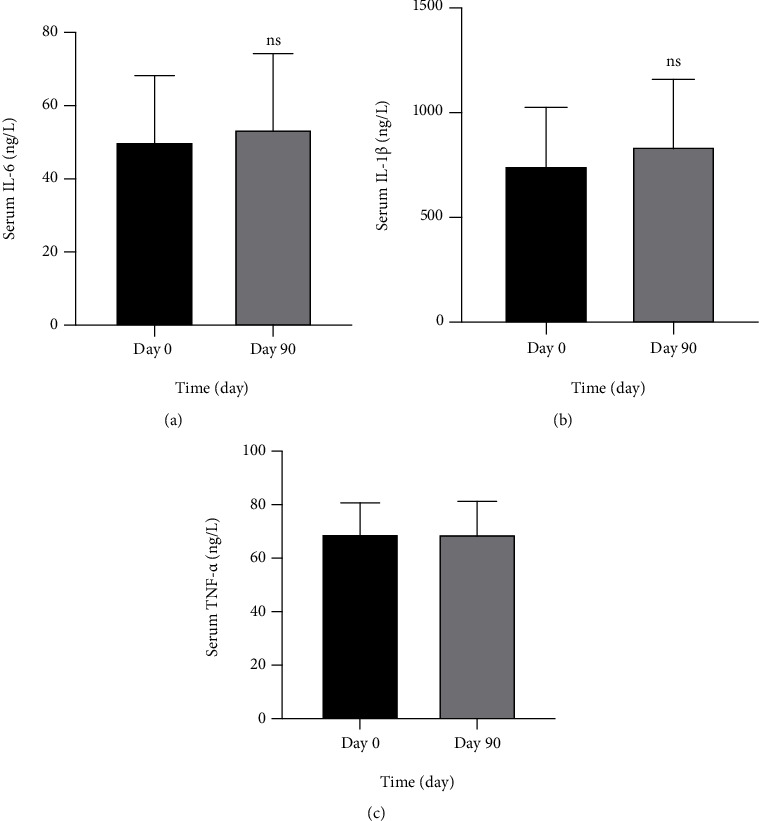
Effect of resveratrol on serum level of IL-6, IL-1*β*, and TNF-*α* (a–c) in patients with knee OA using resveratrol for 90 days. ns: nonsignificantly different comparison of posttreatment with the baseline (pretreatment) using paired *t*-test (*P* > 0.05), *n* = 28.

The effects of resveratrol on the serum level of IL-6, IL-1*β*, and TNF-*α* changes after 90 days of using resveratrol were not significant as shown in Figures 5(a)–5(c), respectively.

### 3.4. Safety of Resveratrol on Hematological Indices with Liver and Kidney Function

Short-term use of resveratrol in patients with knee OA was not associated with hepatic, renal, and hematological parameters. There was a nonsignificant change between the baseline data and 90-day records, as shown in Table 2.

## 4. Discussion

The principal findings of the current study suggest that oral administration of resveratrol as monotherapy for three months could improve knee pain, OA symptoms, stiffness, patient activity, clinical status, and quality of life which has been reflected by KOOS subscale scores and VAS scale for pain. Further, the use of resveratrol was well tolerated and it was not associated with major side effects. Nonsignificant reduction in the collagen network breakdown product (Coll 2-1) biomarker was observed, and aggrecan, a degradation of products of articular cartilage, showed a significant increase in its serum level after 90-day treatment. Many studies emphasized on the role of Coll 2-1 and aggrecan as specific biomarkers for OA [16, 17]. These biomarkers have been verified in animal studies [18], in healthy individuals (i.e., without OA) [19], and in OA patients [20].

Aggrecan, the major proteoglycan in articular cartilage, is essential for compressibility and elasticity of the cartilage. In joint diseases, aggrecan is proteolyzed mainly by matrix metalloproteases (MMPs) and aggrecanases and the released aggrecan fragments from cartilage can be measured in synovial fluid, blood serum, and urine [21].

This study was the first clinical trial that used resveratrol as a monotherapy with the aim of amelioration of pain and preventing the destruction of articular cartilage via Coll 2-1 and aggrecan serum level follow-up. In the present study, the level of these biomarkers did not change in favor of protection of OA progression; in contrary, the level of aggrecan, a specific cartilage biomarker, was increased. The potential hypothesized effect of resveratrol in the present study was not consistent with the earlier study that was reported by Elmali and colleagues in a rabbit model of OA, where there was a significant reduction in the magnitude of cartilage tissue destruction and proteoglycan loss following the administration of intra-articular injections of resveratrol [22].

Similarly, in another *in vivo* study, after intra-articular injections of resveratrol, the expression of type II collagen was preserved; however, the expression of inducible nitric oxide synthase and matrix metalloproteinase-13 was reduced in OA cartilage. The findings of that study also concluded that resveratrol significantly prevented the destruction of OA cartilage by silent information regulator 2 type 1 (SIRT1) activation; eventually, the expression of hypoxia-inducible factor 2*α* (HIF-2*α*) and catabolic factors will be inhibited [23].

Further, in an animal model of obesity-associated OA, oral resveratrol was partially inhibited or delayed the development of OA by decreasing body weight, reducing degradation of type II collagen, and suppressing chondrocyte apoptosis [24].

Many *in vivo* studies have emphasized the protective and regenerative property of resveratrol in OA by exerting a protective effect against matrix degradation and inflammation [25]. The findings of our study were not in line with these observations. The treatment-refractory response of resveratrol upon the improvement of cartilage-specific biomarkers in the current study can be explained by the fact that the enrolled patients in the present study were not in the early stage of osteoarthritis; the duration of this degenerative disease was long enough (more than 2.5 years) that makes it not remarkably regenerated in three months.

Therefore, during the early stages of OA, it would be possible to delay the degradation process through production of aggrecan and inhibiting its destruction as evidenced by Roughley and Mort in their review [26].

We showed previously that adjunct resveratrol supplementation with NSAID reduces pain and inflammation reflected as a significant reduction of serum levels of the proinflammatory markers including IL-6, IL-1*β*, and TNF-*α* in patients with knee osteoarthritis [12]. However, the effect of resveratrol monotherapy in the present study on serum level of proinflammatory cytokines changes was not significant. This can be explained by the fact that combination of resveratrol with the NSAID optimized the dose and provided a synergetic effect of resveratrol, while in the case of the present study, the dose for achieving the effective inhibitory action on those proinflammatory cytokines may not be adequate; also, the short-term therapy of resveratrol may be another reason for this response. It is obvious that inflammatory factors interfere with aggrecan homeostasis by decreasing its synthesis and increasing its catabolism through the upregulation of matrix-degrading enzymes, such as MMPs and aggrecanases [27].

Furthermore, evidence from preclinical studies (*in vitro* and *in vivo*) indicated that IL-1*β* and TNF-*α* are the predominant cytokines involved in the initiation and progression of articular cartilage destruction. IL-1*β*, besides its catabolic activity, inhibits proteoglycan synthesis in cartilage, a process observed in the early stages of OA [28].

In our study, the serum level of these inflammatory cytokines was not modified by 90-day treatment of oral resveratrol; eventually, the destructive effect on articular cartilage may persist and the level of Coll 2-1 and aggrecan remains high.

However, a surprisingly significant improvement in knee pain measured by VAS and KOOS scale has been observed. Additionally, KOOS subscales for functional and activity status were also dramatically improved. The significant clinical improvement in the symptoms and stiffness was predicted because antinociceptive and anti-inflammatory effects of resveratrol can be obtained even within short-term treatment as reported by our previous reports and pilot interventional study on the efficacy of coadministration of resveratrol with meloxicam in patients with knee osteoarthritis [29, 30]. Additionally, other studies demonstrated weak or no significant correlations between aggrecan levels and any of the relevant clinical presentations of OA such as morning stiffness, knee pain, local knee warmth, and other symptoms expressed by Lequesne functional index and Western Ontario and McMaster Universities Arthritis Index (WOMAC) score. However, it was positively correlated with age, body mass index, disease duration, plain X-ray, and MRI scores [31, 32].

The present study also elaborated on the safety issue of resveratrol by investigating liver and renal function tests and hematological indices. The results showed that resveratrol was safe and tolerable at the studied oral dose of 500 mg per day; the present study was consistent with the findings obtained from other follow-up studies that only mild-to-moderate gastrointestinal side effects have been reported in participants who consume more than 1,000 mg/day of resveratrol for up to 29 consecutive days [33] and mild diarrhea was also reported in six out of eight individuals who consumed 2,000 mg of resveratrol twice daily for two periods of eight days in an open-label and within the subject-control study [34].

The current study has some limitations such as the duration of intervention was short, the sample size was relatively small, and there was absence of a control group. However, the strong point is that it was the first clinical trial that used resveratrol as a monotherapy in patients with knee OA with the observation of cartilage-derived biomarkers.

## 5. Conclusion

In conclusion, the present study reported for the first time that oral administration of resveratrol as a monotherapy provides a remarkable improvement in clinical status in patients with knee osteoarthritis but has no significant effect on Coll 2-1 and aggrecan turnover. Further studies are necessary to confirm our results definitively in a randomized clinical trial with a larger sample size and different doses of resveratrol using more specific cartilage-derived macromolecules.

## Figures and Tables

**Table 1:** Demographic data and basic characteristics of the participants treated with resveratrol monotherapy.

Parameters	Resveratrol *n* = 28
Gender	
Male *n* (%)	8 (28.5)
Female *n* (%)	20 (71.4)
Age (year) ± SD	55.96 ± 7.67
Bodyweight (kg) ± SD	82.75 ± 15.83
Duration of knee osteoarthritis (year) ± SD	2.94 ± 2.55
Knee osteoarthritis grade	
Grade I *n* (%)	14 (50)
Grade II *n* (%)	14 (50)
Baseline total KOOS score ± SD	45.69 ± 12.74
Baseline VAS − 100 (mm) ± SD	74.32 ± 8.36
Associated diseases *n* (%)	
Hypertension	8 (28.5)
Diabetes mellitus	3 (10.7)

The results are expressed as mean ± SD (standard deviation) for quantitative variables and as frequency (numbers and percent) for categorized variables. VAS: Visual Analog Scale; KOOS: Knee injury and Osteoarthritis Outcome Score.

**Table 2:** Liver and renal function test parameters and hematological indices in patients with knee OA treated with resveratrol monotherapy for 90 days.

Parameters	Resveratrol *n* = 28		*P* value
	Day 0	Day 90	
Serum GOT (U/L)	21.65 ± 8.32	23.5 ± 8.46	0.14
Serum GPT (U/L)	19.59 ± 7.93	25.07 ± 21.9	0.1
Serum ALP (U/L)	101.1 ± 29.5	98.75 ± 24.97	0.55
Serum urea (mg/dL)	32.3 ± 5.8	32.71 ± 9.35	0.81
Serum creatinine (mg/dL)	0.8 ± 0.174	0.86 ± 0.2	0.048∗
Hb (g/dL)	13.59 ± 1.53	13.68 ± 1.43	0.7
Hct (%)	41.12 ± 4.171	40.61 ± 3.8	0.191
RBC count × 10^6^ (cells/*μ*L)	4.928 ± 0.56	4.9 ± 0.47	0.82
WBC count × 10^3^ cells/*μ*L	6.793 ± 1.59	6.63 ± 1.69	0.455
Platelet count × 10^9^ cells/L	212.4 ± 48.29	217.7 ± 61.5	0.66

Values are expressed as mean ± SD; *n*: number of patients; ^∗^significantly different from the baseline (paired *t*-test, *P* < 0.05); GOT: glutamate oxaloacetate transaminase; GPT: glutamate pyruvate transaminase; ALP: alkaline phosphatase; Hb: hemoglobin; Hct: hematocrit; RBC: red blood cell; WBC: white blood cell.

## Data Availability

All underlying data supporting the result of the study will be available.
